# Randomização Mendeliana na Fibrilação Atrial

**DOI:** 10.36660/abc.20240682

**Published:** 2024-11-21

**Authors:** Protasio Lemos Da Luz

**Affiliations:** 1 Instituto do Coração do Hospital das Clínicas da Faculdade de Medicina da Universidade de São Paulo São Paulo SP Brasil Instituto do Coração do Hospital das Clínicas da Faculdade de Medicina da Universidade de São Paulo, São Paulo, SP – Brasil

**Keywords:** Mendeliana, Fibrilação Atrial, Microbiota

A microbiota intestinal (MI) é um importante mediador para diversas doenças, como diabetes, aterosclerose, hipertensão arterial, obesidade, câncer e doenças neuropsiquiátricas, incluindo Alzheimer, autismo e depressão.^1^ A MI é formada por uma variedade de bactérias, fungos, vírus e arqueas e sua principal função é facilitar a absorção e o metabolismo de alimentos (proteínas, gorduras e carboidratos). Um exemplo das múltiplas ações da MI é a relação bidirecional entre o intestino e o cérebro, o chamado "eixo intestino/cérebro". Além disso, os metabólitos produzidos pela MI são capazes de induzir efeitos localmente ou à distância, o que sugere que o intestino é um órgão endócrino.

A MI é composta por trilhões de células – cerca de 10 vezes mais do que todas as células do organismo humano. Os filos Firmicutes (principalmente espécies de Clostridia) e Bacteroidetes representam cerca de 90% da MI, que também é composta por Actinobacteria, Proteobacteria e Verrucomicrobia.^2^

Mais recentemente, a MI surgiu como um novo fator de risco independente para doenças cardiovasculares.^3–5^ Entre as doenças cardíacas, a fibrilação atrial (FA) também foi associada à disbiose.^6^ As principais causas da FA são hipertensão, doença arterial coronária e insuficiência cardíaca.

Com base em observações experimentais e clínicas, os mecanismos subjacentes que ligam a FA à MI podem ser resumidos brevemente da seguinte forma: A MI produz diversos compostos químicos, incluindo lipopolissacarídeos (LPS), trimetil-amino monóxido (TMAO), ácidos graxos de cadeia curta (SCFAs), ácidos biliares (ABs) e fenilacetilglutamina (PAGLn).^3,7^

Esses produtos agem por meio dos receptores TLR4, GPR43, B_2_ARS e M_2_R tanto em fibroblastos quanto em miócitos, que estimulam os inflamossomos NLRP3 por meio da ativação da NFK-β. A NLRP3 causa a formação de citocinas inflamatórias, ou seja, IL-1β, IL-6 e TNF-α. A ativação dessas vias leva, em última análise, à remodelação estrutural em fibroblastos, remodelação de conexina, remodelação autônoma, remodelação elétrica e remodelação de manuseio de Ca++ em miócitos. O sistema nervoso autônomo exerce um papel fundamental na patogênese e perpetuação da FA.^8,9^ Essas alterações miocitárias levam, em última análise, à reentrada e à atividade desencadeada (manuseio de Ca++), o que causa FA.^10–13^

A disbiose da flora intestinal também prejudica a função de barreira da parede intestinal, agindo sobre proteínas de junção, como conexinas, facilitando os efeitos tóxicos bacterianos no plasma e aumentando a inflamação sistêmica.^12^ ROS são outra ponte entre a MI e a FA, atribuída principalmente à ativação da NLRP-3. Além disso, uma expansão de leucócitos foi observada no miocárdio atrial durante a FA, contribuindo para a liberação de citocinas pró-inflamatórias.^7^ Monócitos e macrófagos são os leucócitos predominantes no tecido cardíaco. Macrófagos podem liberar metaloproteinase de matriz 7, capazes de clivar a matriz extracelular, contribuindo para a remodelação estrutural. Finalmente, a contribuição de micro RNAs (especificamente, miR-177 e miR-204) foi sugerida, com miRNAs influenciando a motilidade cardiovascular e contribuindo para a remodelação dos miócitos.^3^

Em resumo, a relação entre FA e MI é baseada em diversos fatores celulares e metabólicos diferentes. Embora seu papel definitivo ainda não tenha sido completamente esclarecido, a soma das evidências é convincente.

No entanto, nenhuma relação causal direta foi estabelecida entre MI e doença cardiovascular, já que fatores de confusão, como obesidade e estilo de vida, além de pequenas amostras, dificultam tal interpretação.

No estudo de Zhou et al.,^12^ a randomização mendeliana (RM) foi usada para avaliar a possível relação causal entre FA e MI.

Duas amostras da população de ascendência europeia foram analisadas: o consórcio MiBioGen e o Estudos de Associação Genômica Ampla (GWAS) da Twins UK. Assim, um grande número de casos foi incluído: 122, 110 locais de variação distintos originários de 211 táxons em vários níveis (filo, classe, ordem, família, gênero); 45.766 casos de FA e 191.924 controles. Vale mencionar que duas populações diferentes evitam viés de seleção.

As principais suposições sobre RM foram seguidas. Assim, a independência da variável instrumental (VI) foi assegurada pelo uso de Polimorfismo de Nucleotídeo Único (SNP) derivados de grandes GWAS. A pleiotropia horizontal, o fenômeno pelo qual uma VI pode influenciar diferentes resultados independentemente do fator de risco específico estudado, foi considerada e reconhecida quando necessário, mas não interferiu nos resultados principais. Assim, podemos inferir que SNPs específicos de fato se correlacionaram com FA. Uma gama quase completa do reino animal foi analisada, incluindo: filo, classe, ordem, família e gênero.

As principais descobertas incluíram associações positivas e negativas entre AF e determinados componentes da MI. Especificamente, Actinobacteria, Firmicutes, Alloprevotella, Bifidobacterium, Blautia, Eggerthella, Howardella, Ruminococcaceae UCG004 e Ruminococcus1 foram negativamente correlacionados com a FA, enquanto Pasteurellales, Pasteurellaceae, Oxalobacter, Ruminiclostridium5 e Turicibacter foram positivamente associados com a FA.

A plausibilidade biológica precisa ser examinada. Esta é obviamente uma questão intrincada. As descobertas de que determinados componentes da MI podem ser protetores e outros "promotores" são compatíveis com observações clínicas. O fato de alguns pacientes desenvolverem FA e outros não sugere que há diferenças entre eles. Como a FA tem sido consistentemente relacionada à disbiose da MI, a identificação de componentes específicos da MI como protetores ou indutores parece plausível e, de fato, explicaria a razão pela qual alguns pacientes são mais propensos à FA do que outros.

A significância das principais descobertas é considerável. Em vez de estudos observacionais nos quais a confusão é virtualmente impossível de explicar, um estudo de RM oferece uma relação causal direta. Assim, o estudo oferece uma base sólida para decisões médicas e até mesmo possíveis terapêuticas. Um aspecto peculiar é que a MI é composta de muitas espécies. Até que ponto cada uma influencia a prevalência ou persistência da FA permanece um fato desconhecido.

Uma implicação mais prática a possibilidade de interferir com a MI para prevenir a FA ou evitar sua recorrência. Probióticos podem ser uma alternativa, assim como a dieta. Porém, até o momento, não existem evidências conclusivas de que tais intervenções sejam eficazes. Portanto, há desafios consideráveis a serem enfrentados antes que essas novas descobertas possam ser traduzidas em ferramentas objetivas para orientar decisões médicas.
