# Supplementary material for: Randomização Mendeliana na Fibrilação Atrial
Source: Arq Bras Cardiol. 2024 Nov 21;121(11):e20240682. [Article in Portuguese] doi: 10.36660/abc.20240682 (PMC11634291; doi:10.36660/abc.20240682)
Supplement: Translation

# Translation: Mendelian Randomization in Atrial Fibrillation

**DOI:** 10.36660/abc.20240682i

**Authors:** Protasio Lemos Da Luz

**Affiliations:** 1 São Paulo SP Brazil Instituto do Coração do Hospital das Clínicas da Faculdade de Medicina da Universidade de São Paulo, São Paulo, SP – Brazil

**Keywords:** Mendelian, Atrial Fibrillation, Microbiota

Gut microbiota (GM) is an important mediator of several diseases, such as diabetes, atherosclerosis, arterial hypertension, obesity, cancers, and neuropsychiatric diseases, including Alzheimer's, autism, and depression.^1^ The intestinal microbiota is formed by a variety of bacteria, fungi, viruses, and archaea; its main function is to facilitate the absorption and metabolism of foods (proteins, fats, and carbohydrates). One example of the multiple actions of the GM is the bidirectional relationship between the intestine and the brain, the so-called "gut/brain axis." Furthermore, metabolites produced by GM can induce effects locally or at a distance, which suggests that the intestine is an endocrine organ.

The intestinal microbiota is made up of trillions of cells – about 10 times more than all the cells of the human organism. The phyla Firmicutes (mainly Clostridia species) and Bacteroidetes represent about 90% of GM, which is also composed of Actinobacteria, Proteobacteria, and Verrucomicrobia.^2^

More recently, GM emerged as a new independent risk factor for cardiovascular diseases.^3–5^ Among cardiac diseases, atrial fibrillation (AF) has also been associated with dysbiosis.^6^ The main causes of AF are hypertension, coronary artery disease, and heart failure.

Based on several experimental and clinical observations, the underlying mechanisms linking AF and GM can be briefly summarized as follows: GM produces several chemical compounds, including lipopolysaccharides (LPS), trimethyl-amino monoxide (TMAO), short-chain fatty acids (SCFAs), bile acids (BAs), and phenylacetylglutamine (PAGLn).^3,7^

These products act through TLR4, GPR43, B_2_ARS and M_2_R receptors both in fibroblasts and myocytes; they stimulate NLRP3 inflammasomes through NFK-β activation. NLRP3 causes the formation of inflammatory cytokines, i.e., IL-1β, IL-6 and TNF-α. These pathways’ activation ultimately leads to structural remodeling in fibroblasts, connexin remodeling, autonomous remodeling, electrical remodeling and Ca^++^ handling remodeling in myocytes. The autonomic nervous system exerts an overwhelming role in the pathogenesis and perpetuation of AF.^8,9^ These alterations in myocytes ultimately lead to re-entry and triggered activity (Ca^++^ handling), which causes AF.^10–13^

Dysbiosis of intestinal flora also impairs the barrier function of the intestinal wall, acting upon junction proteins such as connexins, facilitating bacterial toxic effects on plasma and adding to systemic inflammation.^12^ ROS is another bridge between GM and AF ascribed mainly to NLRP-3 activation. Furthermore, an expansion of leucocytes has been observed in the atrial myocardium during AF, contributing to the release of pro-inflammatory cytokines.^7^ Monocytes and macrophages are the predominant leucocytes in the heart. Macrophages can liberate matrix metalloproteinase 7, which can cleave extracellular matrix, contributing to structural remodeling. Finally, the contribution of micro RNAs, namely miR-177 and miR-204, has been suggested, with these miRNAs influencing cardiovascular motility and contribute to myocyte remodeling.^3^

In summary, the relationship between AF and GM is based on many different cellular and metabolic factors. Although their definitive role has not yet been completely clarified, the sum of evidence is compelling.

However, no direct causal relationship has been established between GM and cardiovascular disease because confounding factors, such as obesity and lifestyle, in addition to small samples, hamper such interpretation.

In the study by Zhou et al.,^12^ mendelian randomization (MR) was used to assess the possible causal relationship between AF and GM.

Two samples from a population of European ancestry were analyzed: the MiBioGen consortium and the Twins UK registry GWAS study. Thus, a large number of cases was included: 122, 110 distinct variation sites originating from 211 taxa at various levels (phylum, class, order, family, genus); 45,766 AF cases and 191.924 controls. It should be mentioned that two different populations avoid selection bias.

The main assumptions concerning MR were followed. Thus, the independence of the instrumental variable (IV) was assured by the use of SNPs derived from large GWAS. Horizontal pleiotropy, the phenomenon by which an IV can influence different outcomes independent of the specific risk factor studied, was considered and recognized when necessary but did not interfere with the main findings. Thus, we can infer that specific SNPs, in fact, correlated with AF. An almost complete array of animal kingdoms was analyzed, including phylum, class, order, family, and genus.

The main findings were that positive and negative associations between AF and certain MB components were found. Specifically, Actinobacteria, Firmicutes, Alloprevotella, Bifidobacterium, Blautia, Eggerthella, Howardella, Ruminococcaceae UCG004, and Ruminococcus1 were negatively correlated with AF, while Pasteurellales, Pasteurellaceae, Oxalobacter, Ruminiclostridium5, and Turicibacter were *positively* associated with AF.

Biological plausibility needs to be examined. This is obviously an intricate matter. The findings show that certain GM components can be protective and other "promoters" are compatible with clinical observations. The fact that some patients develop AF and others do not suggests that there are differences among them. Since AF has been consistently related to GM dysbiosis, the identification of particular components of GM as protective or inducers seems plausible and in fact, may explain why some patients are more prone to AF than others.

The significance of the main findings is considerable. Rather than observational studies in which confounding is virtually impossible to account for, an MR study offers a direct causal relationship. Hence, it offers a sound basis for medical and even possible therapeutic decisions. A peculiar aspect is that GM is composed of many species. To which extent each one influences the prevalence or persistence of AF remains unknown.

A more practical implication is whether it is possible to interfere with GM in order to prevent AF or avoid recurrence. Probiotics could be an alternative as well as a diet. But so far, there is no conclusive evidence that such interventions are effective. Thus, there are considerable challenges to be addressed before these discoveries can be translated into objective tools to guide medical decisions.
